# Enhanced bioethanol production by evolved *Escherichia coli* LGE2-H in a microbial electrolysis cell system

**DOI:** 10.1186/s40643-023-00717-5

**Published:** 2024-01-03

**Authors:** Cong Wang, Dongdong Chang, Qi Zhang, Zhisheng Yu

**Affiliations:** 1 College of Resources and Environment, University of Chinese Academy of Sciences, 19 A Yuquan Road, Beijing, 100049 People’s Republic of China; 2 RCEES-IMCAS-UCAS Joint-Lab of Microbial Technology for Environmental Science, Beijing, 100085 People’s Republic of China

**Keywords:** Adaptive evolution, *Escherichia coli* LGE2-H, Lignocellulosic pyrolysate, Microbial electrolysis cell

## Abstract

Lignocellulose pretreated using pyrolysis can yield clean energy (such as bioethanol) via microbial fermentation, which can significantly contribute to waste recycling, environmental protection, and energy security. However, the acids, aldehydes, and phenols present in bio-oil with inhibitory effects on microorganisms compromise the downstream utilization and conversion of lignocellulosic pyrolysates. In this study, we constructed a microbial electrolysis cell system for bio-oil detoxification and efficient ethanol production using evolved *Escherichia coli* to overcome the bioethanol production and utilization challenges highlighted in previous studies. In electrically treated bio-oil media, the *E. coli*-H strain exhibited significantly higher levoglucosan consumption and ethanol production capacities compared with the control. In undetoxified bio-oil media containing 1.0% (w/v) levoglucosan, *E. coli*-H produced 0.54 g ethanol/g levoglucosan, reaching 94% of the theoretical yield. Our findings will contribute to developing a practical method for bioethanol production from lignocellulosic substrates, and provide a scientific basis and technical demonstration for its industrialized application.

**Graphical abstract:**

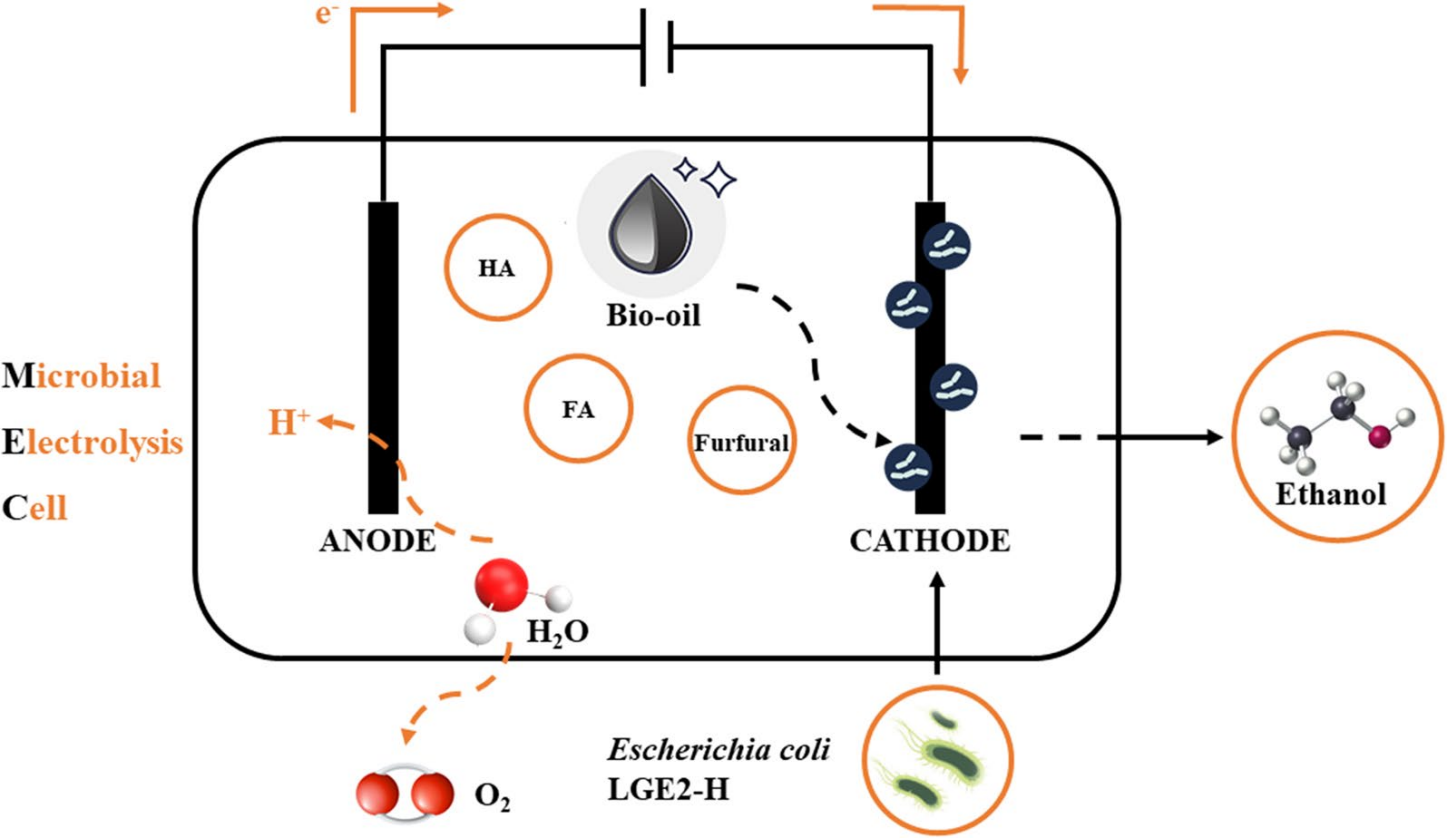

## Introduction

With the increasing depletion of fossil fuel resources and rises in CO_2_ emissions and fuel prices, the development of environmentally sustainable and economically viable alternative sources of renewable energy remains essential (Keasling et al. 2021). After oil, coal, and natural gas, biomass is the fourth largest global source of energy and a vital contributor to the international energy transformation (Huang et al. 2020). To reduce environmental pollution and achieve zero carbon emission, the conversion of biomass to biofuels is an innovative approach that has attracted considerable attention (Saravanan et al. 2022). In this regard, lignocellulose, an abundant, biorenewable, sustainable bioresource, represents a particularly valuable alternative to the raw feedstock to produce biofuels. Indeed, lignocellulosic biomass conversion has become the primary approach to alleviating energy problems.

Microbial bioprocessing of lignocellulosic biomass to produce bioethanol is considered a sustainable blueprint for reducing the depletion of energy reserves and shrinking the carbon footprint. Currently, bioethanol is used extensively worldwide, and Grand View Research, Inc. has reported that the size of the global ethanol market could reach $115.65 billion by 2025, expanding at a compound annual growth rate of 6.7% (Liang et al. 2020). Typically, the raw materials used to produce bioethanol include starch, sugar, and biomass. Although the technologies associated with the use of starch and sugar as raw materials are well-developed and have been extensively used, these methods rely on grain as a raw material, which could exacerbate any future food crises. Consequently, the ethanol derived from the fermentation of lignocellulosic biomass can significantly relieve the pressure on food supplies and maximize the value of waste products (Romans-Casas et al. 2023).

Lignocellulosic biomass comprises cellulose, hemicellulose, and lignin; despite its compact structure, decomposition technologies are necessary to facilitate its biotechnological conversion. In this regard, although chemical and enzymatic hydrolyses are established methods, rapid pyrolysis offers a promising alternative, the resulting primary product of which, referred to as bio-oil, is an energy-rich and readily transportable liquid (Arnold et al. 2017). However, several of the constituents of bio-oil have previously been reported to adversely influence microbial growth, among which the organic acids, aldehydes, and phenolic compounds produced following the pyrolysis of lignocellulosic biomass can inhibit the growth and metabolism of microorganisms, thereby reducing the yield, potency, and productivity of bioethanol fermentation. However, despite ongoing efforts to limit the amounts and types of inhibitors produced during pyrolysis, economically viable processes have yet to be developed (Tao et al. 2022). In the long term, the biological detoxification method, which relies on the “purification” function of microorganisms, is environmentally friendly and cost-effective, and can significantly improve the overall utilization and conversion rates of bio-oil (Wang et al. 2013).

Notably, to date, no fermentation pathway that can utilize undetoxified bio-oil has been identified (Chang et al. 2021). Accordingly, in this study, we sought to use the bioelectrochemical method of microbial electrolysis to enhance the performance of evolutionarily adaptive *Escherichia coli* in the production of ethanol and facilitate direct fermentation of undetoxified bio-oil. Microbial electrolysis cell (MEC) technology is a combination of biological fermentation and electrochemical technologies (Kurgan et al. 2019) that has shown promising applications in waste treatment and bioenergy production technology (Sharma et al. 2022), and has been extensively used in the treatment and preparation of bioenergy, including solvents and alcohols (Prévoteau et al. 2020; Zhen et al. 2017). Cell technology can be used to enhance the resistance of bacterial strains to inhibitors or promote the conversion and ethanol fermentation capacities of strains (Park et al. 2018; Pandit and Mahadevan 2011), thereby reducing raw material processing costs, improving ethanol production efficiency, and facilitating the one-step fermentation of lignocellulose bio-oil to bioethanol. Accordingly, this has significant practical application.

In addition, the previously constructed *E. coli* LGE (a genetically engineered strain), which can utilize levoglucosan (the primary component of bio-oil) to produce ethanol, has been used as a starting strain. It was initially subjected to adaptive evolution under the selection pressure of inhibitors to obtain two evolved strains, namely *E. coli*-H and *E. coli*-L (different adaptive generations), with stable phenotypes and strong resistance to the inhibitory environment of bio-oil. In this study, we constructed an MEC system for pyrolysis, oil detoxification, and efficient ethanol production using these adaptively evolved *E. coli*-H and *E. coli*-L strains to evaluate the enhancement of bioethanol production. This method can be used to directly perform biological detoxification, which is essential for the efficient production of ethanol and may have important implications for biomass energy production.

## Methods

### Bio-oil fractionation

The bio-oil used for this study was prepared using untreated waste cotton in our laboratory (the Joint-Lab of Microbial Technology for Environmental Science, Beijing) with apparatus consisting of four main parts: a tubular furnace pyrolysis reactor, an electrical heater, a temperature controller, and condenser and vacuum system (Chang et al. 2015). The characteristics of the bio-oil are listed in Table 1. In addition to levoglucosan, there are numerous other compounds present in bio-oil, including formic acid, acetic acid, furfural, 2-furylmethyl ketone, 5-methylfurfural, and 4-methyl-2(H)-furanone, all of which are potential inhibitors (Chang et al. 2015).

**Table 1:** Selected characteristics of the raw bio-oil used in this study

Pyrolysis oil properties	Value	Elemental content	Value
Water content (g/L)	970.0	Carbon	32.13
Density (g/mL)	0.974	Nitrogen	0.37
pH	2.3	Hydrogen	8.45
Levoglucosan (g/L)	100	Oxygen	56.91
Furfural (g/L)	3.0		
2-Furylmethyl ketone (g/L)	4.0		
5-Methylfurfural(g/L)	3.0		
4-methyl-2(H)-furanone (g/L)	2.5		
Acetic acid (g/L)	2.0		
Formic acid (g/L)	1.0		

### Strains

The three *E. coli* strains used in this study are listed in Table 2, among which the levoglucosan-utilizing and ethanol-producing strain *E. coli* LGE2 was previously constructed by us (Chang et al. 2021). The evolved strains *E. coli*-L and *E. coli*-H were used as the production strains (Palazzolo and Garcia-Perez 2022). *E. coli*-L has evolved from *E. coli* LGE2 through 302 generations, and *E. coli*-H has evolved from *E. coli*-L through 72 generations.

**Table 2:** *Escherichia coli* strains used in this study

Strains	Description	Source
*E. coli* LGE2	*F*^*−*^* omp*T *gal dcm lon hsd* SB (r*B*^*−*^m*B*^*−*^) λ(DE3) *lgk pdc adh Amp*^*r*^* Cm*^*r*^	Laboratory collection
*E. coli*-L	Evolved from *E. coli* LGE2 over 302 generations	This study
*E. coli*-H	Evolved from *E. coli*-L over 72 generations	This study

Underlined text represents heterologous genes

### Growth conditions

The *E. coli* strains were grown in Luria broth medium (per liter: peptone, 10 g; yeast extract, 5 g; and NaCl, 5 g), and bio-oil-based M9 minimal medium (per liter: Na_2_HPO_4_, 7.10 g; KH_2_PO_4_, 3.00 g; NaCl, 0.50 g; NH_4_Cl, 1.00 g; MgSO_4_, 0.49 g; CaCl_2_, 14.7 mg) and 10% bio-oil were used for ethanol fermentation. Media were supplemented with ampicillin (100 mg/L; Solarbio, China), chloramphenicol (34 mg/L; Solarbio, China), and isopropyl β-d-1-thiogalactopyranoside (1 mmol/L; Solarbio, China) at final concentrations of 100 µg/mL, 34 µg/mL, and 0.06 mM, respectively.

### Bioelectrochemical reactor design

**Fig. 1:**
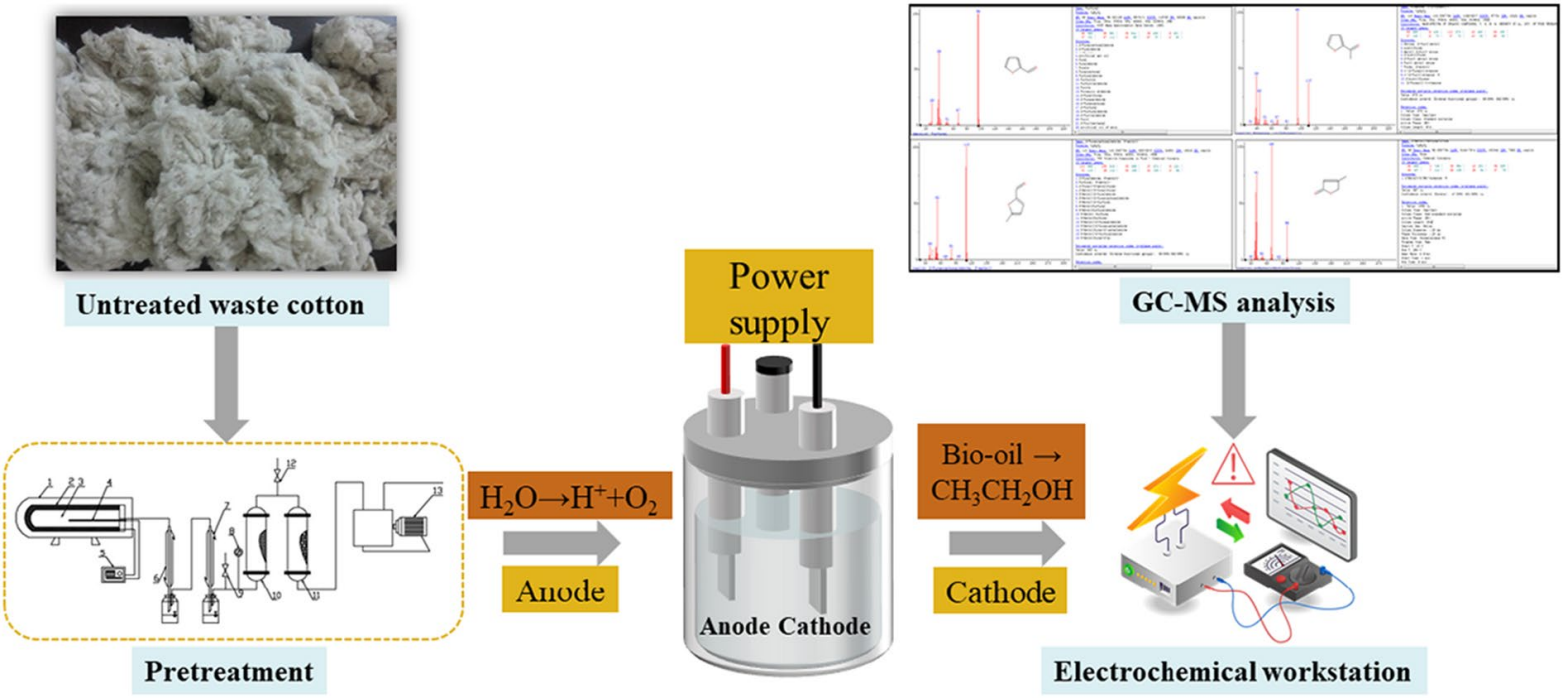
*Escherichia coli* microbial electrolysis cell reaction chamber constructed and used in this study

The bioelectrochemical reactors (BERs) used in this study, fabricated with double-layer jackets of plexiglass, were designed to have a total/working volume of 3.5 L (Fig. 1). The inner chamber (15 × 20 cm) was closed to achieve microaerobic microenvironments intended for bioethanol production. The outer layer (20 × 20 cm) was filled with water to monitor leaks and maintain a constant reaction temperature. Side arms facilitated the sampling of media using a syringe, and a drain port located at the bottom of the reactor enabled the flushing out of reactor contents whenever required. The BER was operated in batch mode at 30 ℃, with stirring performed using a magnetic stirrer (150 rpm) to ensure an even distribution of the reactants.

A 50 × 20 cm carbon cloth was nipped using the platinum electrode holders, and a graphite felt coupon connected to a titanium wire was submerged into the fermentation medium to serve as the working electrode during electrolysis (Yu et al. 2021). In the three-electrode reactor, the electrodes were linked with the potentiostat for electric control and measurements.

### Microbial electrolysis reaction setup

According to the concentration of levoglucosan in the bio-oil, 10% (v/v) bio-oil was added to M9 minimal medium as the sole carbon source, resulting in a final levoglucosan concentration of 10 g/L in the fermentation medium with a pH of 3.1. The *E. coli* strains were pre-cultured in Luria broth medium (pH 7.0) in 250-mL flasks incubated overnight at 30 ℃ with shaking at 150 rpm. Thereafter, 10 mL of these starter cultures were inoculated into each three-electrode BER, which contained 1 L of fresh bio-oil and M9 minimal medium for microbial electrolysis under microaerobic conditions. The initial potential of the working electrode (cathode) was controlled at − 650 mV (Harrington et al. 2015), the sampling time was set at 3 days, and the sensitivity (A/V) was set at 1.0 × 10^–3^. The BER was connected to a DC-power supply (GW INSTEK GPE-4323C) operated under constant voltage conditions, whereas the voltage was set to a maximum of 1.0 V and carefully monitored (Kondaveeti and Min 2015). The experiments were conducted with the addition of 0.5 mM of neutral red (Aladdin), an exogenous redox mediator.

Batch experiments were simultaneously conducted in four groups, for which the negative control setups contained no electrodes and were not connected to a potentiostat. For each sampling time point during 56-h cultivation, a 5-mL culture sample was withdrawn at 4-h intervals to measure the concentrations of levoglucosan, ethanol, formic acid, acetic acid, and furfural. All fermentation tests were performed in triplicate.

### Analytical procedures

#### Growth rate measurement

The growth rates of cultures were assayed by measuring the optical density (600 nm) of fermentation cultures in 10-mm glass cuvettes using a UV 759 spectrophotometer (Shanghai Yoke Instrument Co., Ltd., Shanghai, China).

#### Chromatographic analysis

The concentrations of levoglucosan and ethanol in the fermentation culture were detected via high-performance liquid chromatography (LC-20AT; Shimadzu Corp.) using an ion-exchange column (Transgenomic ICSep ICE-ION-300; 300 mm × 7.8 mm) in conjunction with a refractive index detector (RID-10A; Shimadzu Corp.). As a mobile phase, we used 0.0085 N H_2_SO_4_ at a flow rate of 0.4 mL/min, with the column temperature being maintained at a constant 67 ℃. The concentrations of formic acid, acetic acid, and furfural in the fermentation culture were determined using a C18 column (250 mm × 4.6 mm, 5 µm particle size; TOSOH Corp.) employing an UV absorbance detector (SPD-20A). As a mobile phase, we used H_2_O/methanol (10/90, v/v) at a flow rate of 1 mL/min and an operating temperature of 40 ℃. The injection volume was 20 µL.

#### Bioelectrochemical analysis

Control, measurement, and analysis of the electric parameters were conducted using a 660E eight-channel potentiostat (Huakeputian Instruments Co., Ltd., Beijing, China). The bioelectrochemical behavior of *E. coli* during bioethanol production was studied using CVs by applying a potential ramp to the working electrode (anode) over a scan range from − 1.0 to + 1.0 V at a scan rate of 0.1 V s^−1^ against an Ag/AgCl (S) reference electrode (Sugnaux et al. 2016). The current delivered to the fermentation medium was monitored using a custom-built potentiostat; a negative current indicated the delivery of electrons to the fermentation medium (cathodic current).

### Statistical analysis and calculations

The Student’s *t*-test function of Origin 9.0 software was used to analyze statistical differences between the data obtained in the aforementioned experiments. Two-tailed *t*-tests were performed assuming an unequal variance. Differences indicated by a *p value* < 0.05 were considered to suggest a clear trend, whereas *p* < 0.01 and *p* < 0.001 denoted significant and very significant differences, respectively.

The sampling was set at 56 h from the start of electrolysis. The Coulomb efficiency for the formation of ethanol (FE_CH3CH2OH_) was calculated as follows:

$$ {\text{FE}}_{{\text{CH3CH2OH}}} \; = \;\frac{{n_{CH3CH2OH} \times n \times F}}{{\int_0^t {I {\text{dt}}} }}, $$

where *n*_CH3CH2OH_ represents an increase in the moles of ethanol harvested in the MEC, *n* represents the number of electrons required for the formation of one molecule of ethanol from levoglucosan (*n* = 4), *F* represents the Coulomb constant (96,485  C/mol of electrons), and *I* is the circuit current.

## Results and discussion

### Enhanced bioethanol productivity using an MEC system

**Fig. 2:**
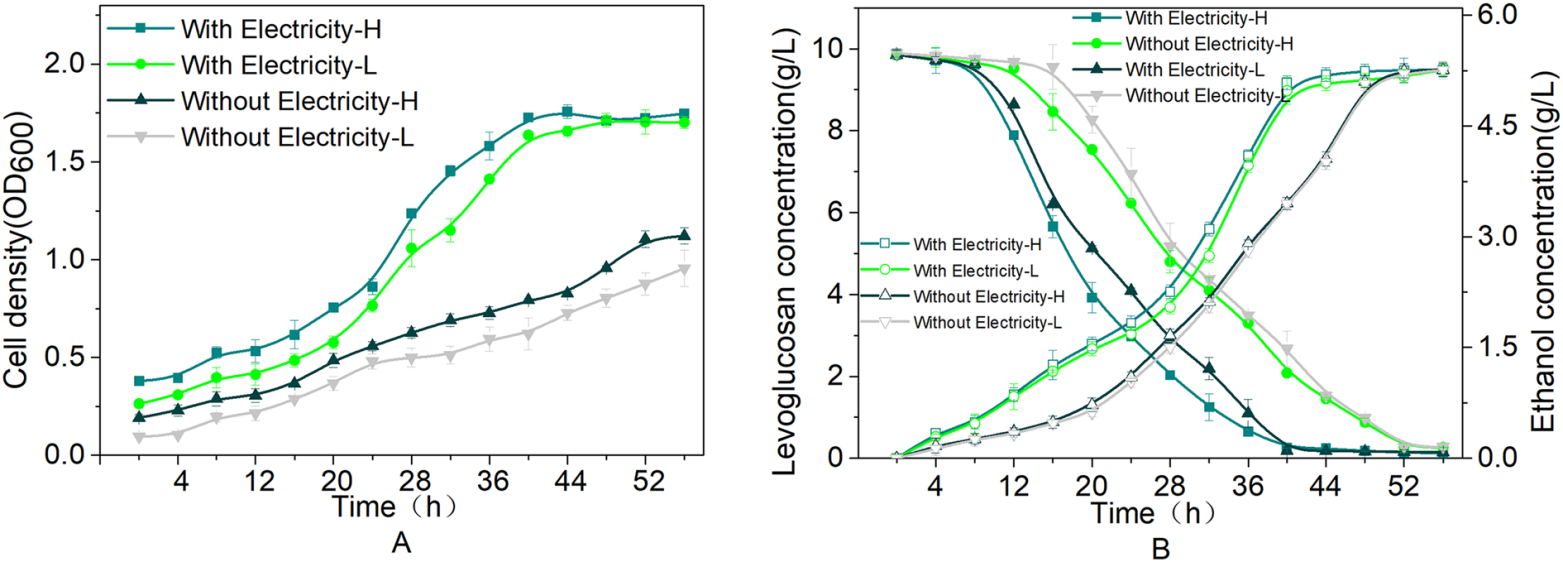
Cell growth and ethanol fermentation of the evolved *E. coli*-H and *E. coli*-L strains in a microbial electrolysis cell system. **a** Cell growth. **b** Levoglucosan consumption and ethanol production

In this study, we constructed an MEC system, in which the cathode was employed as a working electrode that delivered electrons to electron carriers, which in turn transferred these electrons to *E. coli* in the fermentation medium. The other two electrodes in the MEC system were the Ag/AgCl electrode, used as a reference electrode, and the anode, used as a counter electrode. As a model electron carrier, we used neutral red. The cell membranes of *E. coli* contain protein complexes that facilitate the input of electrons transferred from electron carriers, and within the cell, these electrons are then transferred to biological carriers and converted into reducing power for biosynthetic reactions. As an indicator of MEC system performance, we measured the amount of bioethanol produced from 10 g/L levoglucosan used as a reaction substrate. Initially, we investigated the typical batch fermentation process of *E. coli*-H and *E. coli*-L in the reactors without applying a potential. As shown in Fig. 2A, cell growth increased from 16 h, and after 44 h, the cell density had stabilized. Based on constructed levoglucosan consumption and product generation curves, we determined that 9.7 g/L of levoglucosan was consumed within 48 h, with the corresponding production of 5.2 g/L bioethanol, thereby representing a bioethanol yield and productivity of 0.53 g/g and 0.11 g/L/h, respectively. This effective yield of 0.53 g ethanol/g levoglucosan represents ~ 91% of the theoretical yield.

We subsequently examined the effects of electricity on fermentation using the MEC system and found that cell density increased rapidly from 12 h, reaching a maximum OD_600_ of 1.76 after 40 h of cultivation, after which the cell density remained stable. During fermentation, the levoglucosan consumption curve was observed to show a close correspondence to the growth curve. As expected, ethanol production in the electrified MEC system gradually increased and was higher than that in the control group. At the end of the fermentation, 9.8 g/L levoglucosan had been consumed in 44 h, with 5.3 g/L of bioethanol being produced by the *E. coli*-H strain (Fig. 2B). The relative amounts of bioethanol produced were similar to those produced by the control, this yield was obtained in a shorter period. Compared with the unelectrified system, the application of electricity promoted a significant enhancement in the rate of levoglucosan conversion (0.19 g/L/h). These findings indicate a high biomass accumulation and accelerated levoglucosan consumption contributed to the comparatively rapid production of ethanol. Thus, based on the data obtained, the yield and productivity of bioethanol were calculated as 0.54 g/g and 0.19 g/L/h, respectively. *E. coli*-H produces 0.54 g ethanol/g levoglucosan, which is 94% of the theoretical yield, almost exclusively derived from levoglucosan. As shown in Fig. 2B, the ethanol productivity of 0.19 g/L/h achieved with the application of electricity was higher than that of 0.11 g/L/h achieved without electricity. Notably, the bioethanol productivity and yield obtained using the electrified MEC system were 72.7% and 3.3% higher than those measured in the reactors without electricity, and similar results were obtained using the *E. coli*-L strain. These findings indicate that relatively high production and productivity of bioethanol can be achieved using the designed MEC system.

### Comparison of fermentations

Improvements in product yield obtained via electrical enhancement are dependent on the biochemical compounds produced and the substrate utilized (Layton et al. 2011). Maximizing product synthesis involves redirecting the carbon flux and electron transfer to product formation rather than the generation of biomass and establishes an upper limit on the product yield.

**Fig. 3:**
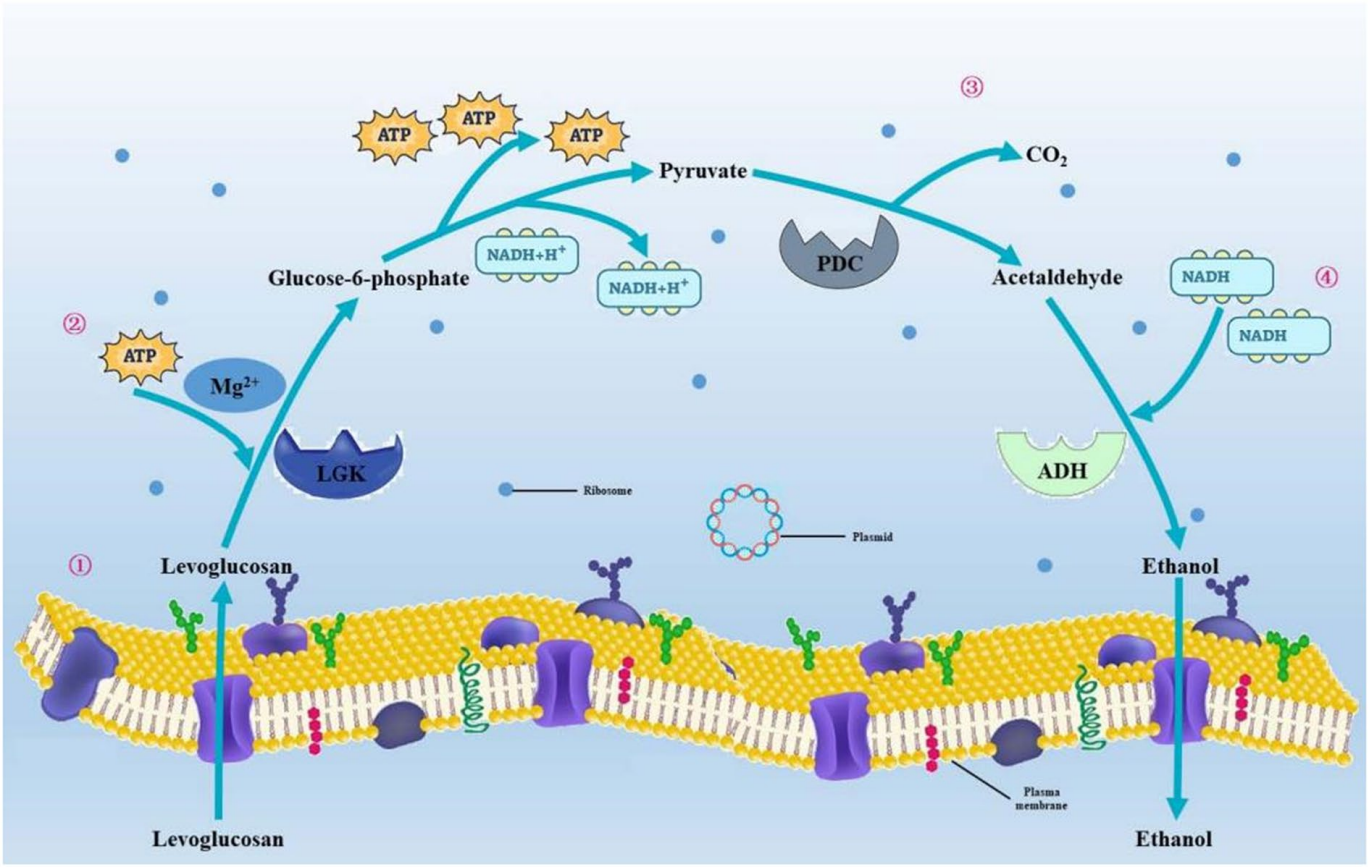
Metabolic pathway for the production of ethanol from levoglucosan. (1) Levoglucosan is transported to the plasma membrane. (2) Levoglucosan is catalyzed by levoglucosan kinase (LGK) to yield glucose-6-phosphate in the presence of Mg.^2+^ and ATP. (3) Pyruvate is transformed to acetaldehyde by pyruvate decarboxylase (PDC). (4) Acetaldehyde is reduced to ethanol by alcohol dehydrogenase (*adh*)

Electrical enhancement can make a significant contribution to improving yields when the reducing power of the substrate is relatively small compared with the product’s degree of reduction. We showed that *E. coli* can ferment levoglucosan to ethanol in the presence of an electrode-based electron acceptor, the process of which involves a complex metabolic pathway (Fig. 3) (He et al. 2016). Levoglucosan is reduced in the presence of levoglucosan kinase to form pyruvate and subsequently bioethanol, with these reactions occurring under the influence of redox electron carriers, such as nicotinamide adenine dinucleotide (NADH) and adenosine-triphosphate (ATP). Furthermore, it has been established that 4 mol of ATP and 2 mol of NADH are required for the conversion of 1 mol of levoglucosan to acetaldehyde, whereas 3 mol of ATP and 2 mol of NADH are consumed in the reduction of 2 mol of acetaldehyde to ethanol.

Notably, metabolic pathways in *E. coli* often contain metabolic nodes. Analysis has established that these nodes are associated with bioethanol production and could be attributed to *E. coli* metabolism. Alcohol dehydrogenase (*adh*) is the enzyme responsible for this conversion, and consequently, the additional NADH generated by reactions at the electrode could induce a shift in *E. coli* metabolism toward pathways that balance the redox potential (Molognoni et al. 2023), thereby resulting in a substantial enhancement in bioethanol productivity.

These data would tend to indicate that electrical enhancement could serve as a means to regulate the flux through *adh* as a response to the NADH generation (Gu et al. 2022), and thus increasing the applied current to *E. coli* would result in an elevated NADH/NAD^+^ ratio (Xie et al. 2022). Consequently, pathways contributing to the regeneration of NAD^+^ would be characterized by higher fluxes (Li et al. 2022), resulting in relatively higher bioethanol productivity. Moreover, we found that the application of electricity clearly stimulated the growth of *E. coli* and markedly diminished the necessary fermentation time. These findings indicate that this bioelectrochemical method can be used to enhance the yields and productivity of bioethanol using the MEC system.

### Inhibitor degradation performance

**Fig. 4:**
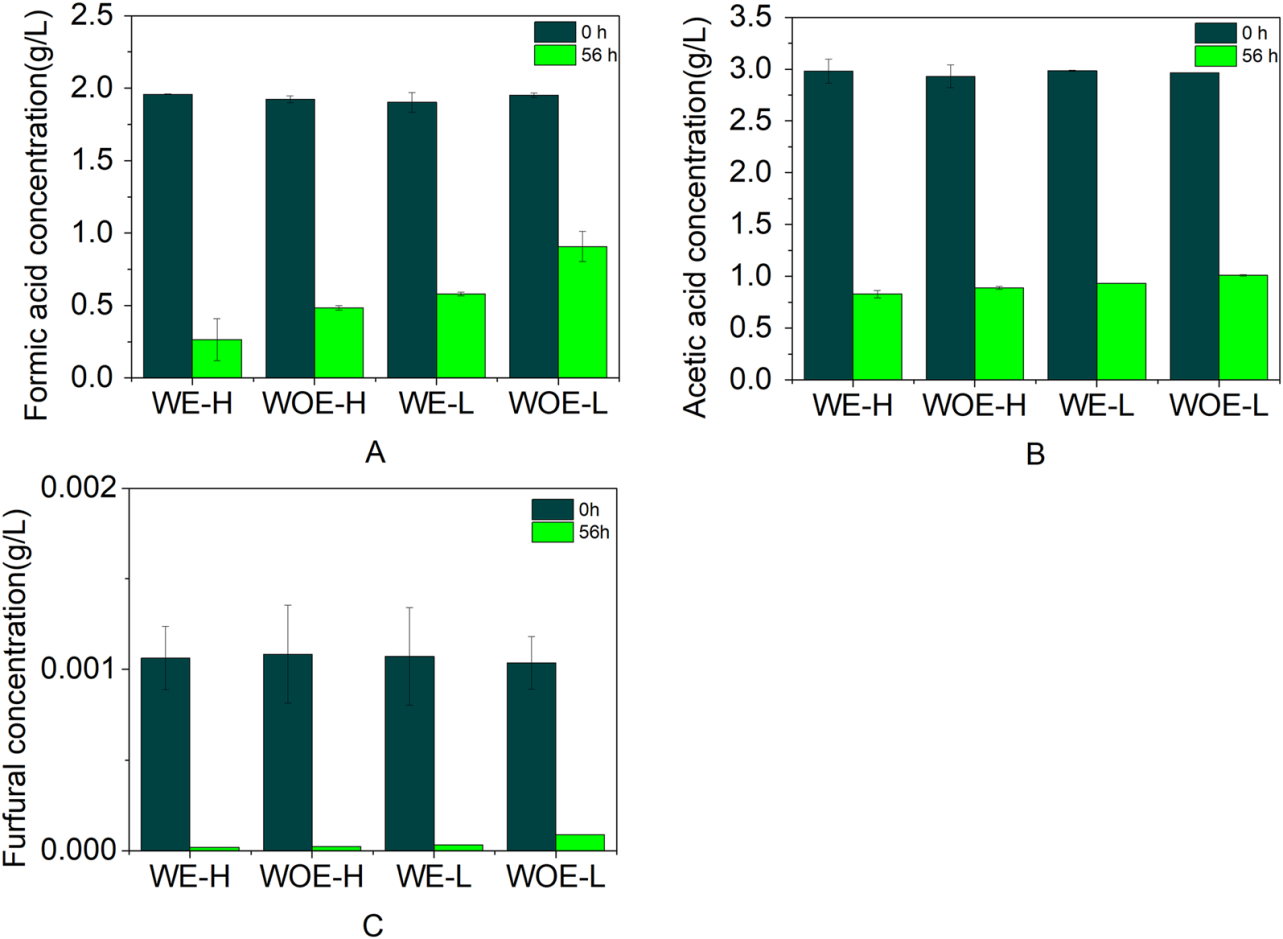
Inhibitor conversion by the evolved *E. coli*-H and *E. coli*-L strains in a bio-oil-based microbial electrolysis cell system. **a** Formic acid, **b** acetic acid, and (**c**) furfural concentrations before (0 h) and after (56 h) fermentation. Conditions: 30 ℃, 200 rpm, pH 3.1. Mean values are presented with bars representing at least two standard deviations

In bio-oil media, both with and without the application of electricity, we detected changes in the concentrations of acetic acid, formic acid, and furfural among the four treatment groups, and observed significant differences in the concentration of these inhibitors after fermentation for 56 h, with the concentrations of all assessed inhibitors declining during fermentation. In the bioelectrochemical group, the concentration of furfural had dropped to approximately 2 × 10^–8^ mg/L at 56 h (Fig. 4), which compares with the value of approximately 8.83 × 10^–8^ mg/L recorded at the same time point in the control fermentation. The lowest final concentration of furfural (1.87 × 10^–8^ mg/L) was detected in the *E. coli*-H fermentation system with applied electricity, which was approximately half that measured in the *E. coli*-H control system. In contrast to furfural, which was almost completely metabolized (> 99%) in the presence of electricity, we detected only slight changes in the concentration of formic acid and acetic acid, with 0.26 and 0.83 g/L remaining, respectively. The estimated conversion of formic acid was between 74 and 83%, whereas that of acetic acid was between 59 and 72%, with total reductions of between 82 and 88% being obtained for aldehyde compounds, thereby demonstrating the significant conversion of the furan aldehyde and acid compounds in the bio-oil. Similar results were obtained in fermentations using *E. coli*-L. These findings thus indicate that microbial electrolysis can promote the efficient conversion of the organic acids and furfural in bio-oil. In addition, we observed a high level of conversion of the quantified aldehyde compounds, as evidenced by the “Total” percentage reduction, which included furfural and 5-methylfurfural. Furthermore, the *p* values obtained for the removal of formic acid, acetic acid, and furfural in the electricity group throughout the experiment were 0.003, thereby indicating that the application of electricity significantly enhanced the degradation of inhibitors, which could be attributable to the fact that *E. coli* can detoxify furfural to less toxic alcohols via an innate degradation pathway, and it is conceivable that this process might have been enhanced by the application of a weak electric current (Kondaveeti and Min 2015; Zeng et al. 2017), and the fact that *E. coli* can use furfural as electron donor (Steinbusch et al. 2010; Speers et al. 2014).

### *E. coli* electrolysis analysis

**Fig. 5:**
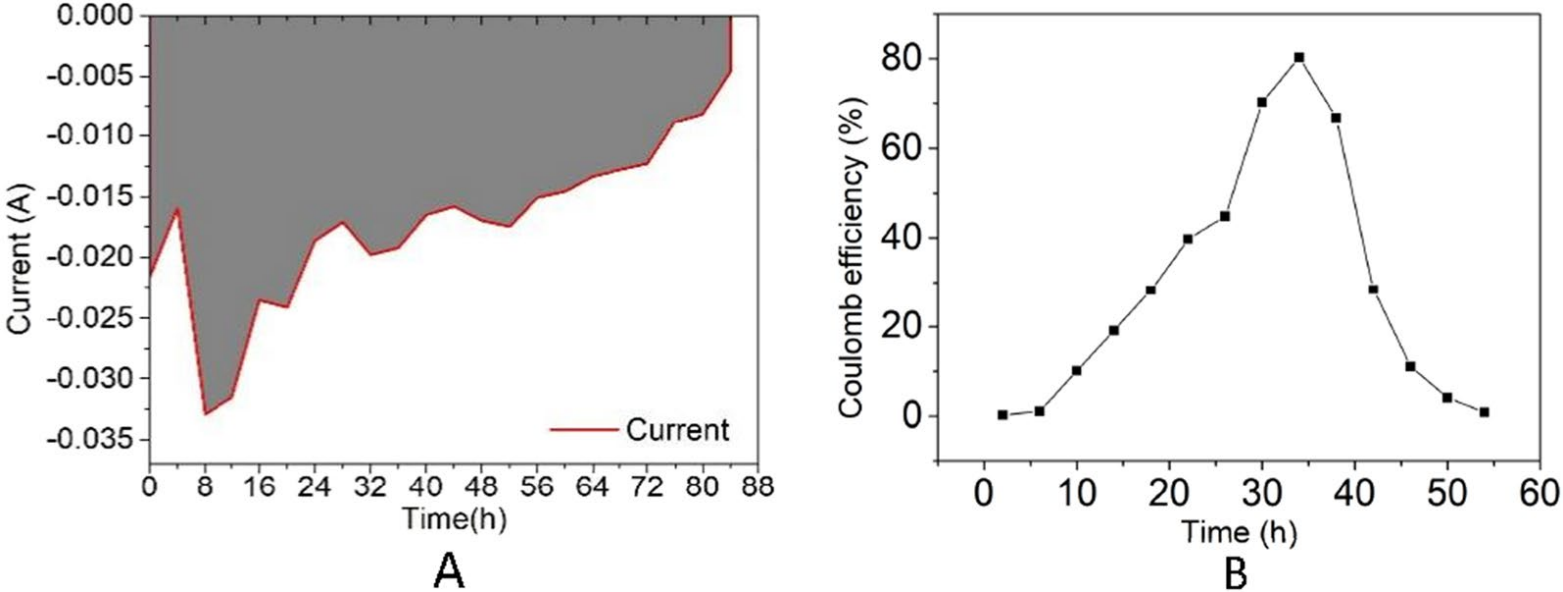
Electrosynthesis profiles for the *E. coli*-H strain growing in bio-oil. **a** Chronoamperometry plot of the current delivered to the fermentation medium. The gray-shaded area represents the area integrated to determine the total charge transferred, (**b**) Coulomb efficiency of the experimental group

Electrolysis during the fermentation of bio-oil by *E. coli* resulted in current changes, with an initial increase in the current after inoculation from 0 to 4 h, which then dropped with continued cell growth from 4 to 8 h, prior to slowly increasing toward that of the background after the cells had stopped growing (Fig. 5). Neutral red functions as a supplementary electron sink that accepts the excessive electrons produced during glycolysis, which are in turn used to regenerate NAD^+^ from NADH, which is beneficial for further glycolysis. Furthermore, the ATP production increased when neutral red was added during the fermentation of *E. coli*, which is also beneficial for the enhancement of electron transfer (Chen et al. 2021).

The application of an electric potential had the effect of stimulating bioethanol production and also induced *E. coli* growth, although the underlying mechanisms have yet to be determined. Notably, this enhancement of active *E. coli* growth by electricity does not necessitate a prior detoxification of the bio-oil, indicating that the pressure resistance of *E. coli* cells was significantly enhanced in response to the applied potential.

The Coulomb efficiency for the production of ethanol in the MEC process represents the ratio of the mass of ethanol liberated by a current to the theoretical mass, as predicted by Coulomb’s law. This measure of efficiency is a key index for evaluating factors such as the effects of side reactions and current leakage. The Coulomb efficiency is calculated on a 4-h interval for the fermentation process, which, for the experimental group, showed a trend of first increasing and then decreasing (Fig. 5). In this study, we found that the Coulomb efficiency of the experimental group peaked at 80.3%, 66.8%, and 28.4% during the course of fermentation from 32 to 36 h, 36 h to 40 h, and 40 h to 44 h, respectively, thereby indicating that external energy is the main source for generating bioethanol during the fermentation process, although gradually declines in the latter stages. These findings indicate that the MEC system used in the present study is functionally efficient and can contribute to increasing the yield of ethanol.

Finally, electro-fermentation can be used to optimize microbial processes and thus make a significant contribution to emerging biomass refinery chains (Martin et al. 2018). We postulate that *E. coli* can directly utilize the electrons derived from applied currents or adjust their metabolic flux in response to different redox conditions (Li et al. 2023). Given the potential value of this approach, the mechanisms whereby the electrical potential and current influence the metabolism and growth of microbial strains in electro-fermentation should be elucidated (Mostafazadeh et al. 2017). Using such MEC systems, the fermentation process can be regulated and optimized to achieve products with high purity and to enhance microbial cell growth and density (Wu et al. 2019).

## Conclusion

In this study, we demonstrated the application of a microbial electrosynthesis process for the fermentative production of ethanol. Using the MEC system, we obtained a productivity of 0.54 g ethanol/g levoglucosan, with almost complete conversion of levoglucosan being observed, reaching 94% of the theoretical yield. Notably, when using an electrified MEC system, the productivity of bioethanol was 72.7% higher than that obtained using the system without the application of electricity. In addition, with respect to potential inhibitory compounds, we obtained maximal furfural and organic acid conversion efficiencies of 99% and 83%, respectively. Moreover, the Coulomb efficiency of the MEC process reached a maximum value of 80.3%. We conclude that the ethanol produced from the integrated process demonstrated in this study can be used to derive fuel from hydrodeoxygenated bio-oil, while reducing lifecycle greenhouse gas emissions.

## Data Availability

The datasets used or analyzed in this study are available from the corresponding author upon reasonable request.

## Abbreviations

*adh*: Alcohol dehydrogenase
ATP: Adenosine-triphosphate
BER: Biochemical reactor
CV: Cyclic voltammogram
MEC: Microbial electrolysis cell
NADH: Nicotinamide adenine dinucleotide
